# Time dependent changes in protein expression induced by intermittent theta burst stimulation in a cell line

**DOI:** 10.3389/fneur.2024.1396776

**Published:** 2024-10-28

**Authors:** Fatima Y. Ismail, Manigandan Krishnan, Richard L. Jayaraj, Gilles Bru-Mercier, Mauro Pessia, Milos R. Ljubisavljevic

**Affiliations:** ^1^Department of Pediatrics, College of Medicine and Health Sciences, UAE (United Arab Emirates) University, Al Ain, United Arab Emirates; ^2^Department of Neurology (Adjunct), Johns Hopkins University School of Medicine, Baltimore, MD, United States; ^3^Department of Physiology, College of Medicine and Health Sciences, UAE (United Arab Emirates) University, Al Ain, United Arab Emirates

**Keywords:** transcranial magnetic stimulation, intermittent theta burst stimulation, N2A cells, temporal dynamics, N-methyl-D-aspartate (NMDA), glutamate receptors, γ-aminobutyric acid (GABA) receptors

## Abstract

**Background:**

Intermittent Theta Burst Stimulation (iTBS), a non-invasive brain stimulation technique, is recognized for its ability to modulate cortical neuronal activity. However, its effects over time and the dynamics following stimulation are less well understood. Understanding the temporal dynamics of iTBS effects is essential for optimizing the timing and frequency of stimulation in therapeutic applications.

**Objective:**

This study investigated the temporal changes in protein expression induced by iTBS in Neuro-2a cells.

**Methods:**

We analyzed protein expression in retinoic acid-differentiated Neuro-2a cells at multiple time points — 0.5, 3, 6, 12, and 24 hours post-iTBS — using Western blot and immunocytochemistry techniques.

**Results:**

Our findings reveal a significant early increase in neurotransmitter receptor subunits, neurotrophic factors, and cytoskeletal proteins within the first 0.5 hour following iTBS. Notably, proteins such as mGLuR1, NMDAR1, GABBR2, and β-tubulin III showed substantial increase in expression. However, the effects of iTBS on protein expression was not sustained at later timepoints.

**Conclusion:**

Our results suggest that iTBS can transiently alter the expression of specific proteins in Neuro-2a cells. Future research should investigate the potential benefits of repeated stimulations within the early time window to refine iTBS interventions, potentially expanding their research and clinical applications.

## 1 Introduction

Transcranial magnetic stimulation (TMS) is a non-invasive brain stimulation technique that induces a magnetic field in a targeted brain region by passing an electric current through a magnetic coil. This method modulates neuronal activity and synaptic plasticity by several cellular and molecular mechanisms, depending on the frequency and pattern of the stimulation. TMS is widely used in both research and clinical settings to assess cortical excitability, induce or inhibit synaptic plasticity, evaluate the integrity of corticospinal tracts (1), map cortical regions (2) and modulate both local and distant cortical circuits (3).

iTBS is a specific form of repetitive TMS where bursts of three pulses at 50 Hz are repeated at intervals of 200 milliseconds (equivalent to 5 Hz) to mimic the natural cortical oscillations at theta rhythms (4). A typical iTBS protocol involves 2-s trains of these bursts, with an 8-s rest period between trains, and a total of 600 pulses administered over approximately 3 min, which makes it more efficient for clinical applications compared to other repetitive TMS protocols. iTBS protocols vary in pulse quantity (600 vs. 1,200 pulses per train), stimulation intensity, duration, and target areas of stimulation (5, 6).

iTBS is shown to enhance cortical excitability and induce long-term potentiation-like effects. It can induce long-lasting neuroplastic changes in corticospinal motor output as measured by motor evoked potential (MEP) (7), enhance motor function post-stroke (8), support recovery after spinal cord injury (9) and treat various neurological and psychiatric disorders, such as depression (10), cognitive dysfunction (11), and dementia (12).

Despite its versatile clinical applications, the precise neuromodulatory mechanisms underlying iTBS and the dynamic nature of its effects on neuromodulation remain partially understood. In particular, there is limited research on how neuronal excitability and plasticity evolve over time following iTBS. The temporal dynamics of the after-effects—specifically, how these effects change at various time points post-stimulation—remain unclear.

To date, only a limited number of studies have explored the after-effects of iTBS at multiple timepoints following stimulation within the same experimental framework (13–15). However, these studies differ significantly in their protocols, models, protein targets, and assessment intervals. For example, Zhu et al. (16), applied electrical stimulation to dorsal root ganglion neurons, focusing on assessing c-Fos and BDNF expression at various timepoints after stimulation. Similarly, other studies investigated the effects of iTBS on SH-SY5Y cells, examining changes in plasticity-related proteins (NTRK2 and MAPK9) at 24 hours post-stimulation (14). Wang et al. (15) explored the dose-dependent effects of iTBS in a Parkinsonian rat model, measuring proteins such as CREB, BDNF, and c-Fos at several intervals: immediately, 1 hour, 24 hours, and 48 hours post stimulation.

Investigating the time-dependent changes in the after-effects can help identify critical windows when cells are most or least responsive to stimulation. This understanding is essential for designing effective treatment protocols, particularly for disorders where the timing of intervention plays a crucial role.

In this study, we aimed to address this gap by investigating the dynamic changes in the expression of neurotransmitter receptor subunits, neurotrophic factors, and cytoskeletal proteins previously reported to be affected by iTBS at five distinct time points following a single session of iTBS (14, 17–19).

We chose to use a 300-pulse iTBS train, deviating from the standard 600-pulse train typically used in clinical settings. This decision was based on two key considerations: first, multiple studies and meta-analyses have reported substantial variability in modulation of cortical excitability and functional connectivity following the conventional 600-pulse protocol (20–22). Second, existing evidence suggests that increasing the number of pulses within iTBS sessions may inversely affect MEP amplitudes (6). The neuromodulatory effects of using fewer than 600 pulses remain largely unexplored. Our aim was to investigate the mechanisms underlying iTBS-induced modulation while minimizing the potential confounding effects of pulse quantity at this initial stage of the study. Additionally, we modified the iTBS protocol to 300 pulses while utilizing the maximum stimulation output of the TMS device, a setting not typically employed in standard clinical iTBS protocols. Although this approach differs from typical clinical protocols, it allows us to approximate the total energy delivered in a way that closely mirrors clinical settings, despite the reduced number of pulses. This adjustment, alongside our primary considerations—addressing variability in protein modulation with standard protocols and the nuanced effects of pulse quantity—was intended to provide a balanced energy input. By doing so, we aimed to isolate and understand the fundamental neuromodulatory mechanisms under conditions comparable to those in clinical settings.

## 2 Methods

### 2.1 Cell culture and differentiation

Neuro-2a (N2A) mouse neuroblastoma cells, obtained from ATCC (CCL-131), were cultured in DMEM supplemented with 10% FBS and 1% penicillin/streptomycin. These cells were incubated at 37°C in a humidified 5% CO_2_ environment. To ensure cellular integrity and avoid senescence, a limit of 24 passages was maintained for the N2A cells.

Differentiation of N2A cells was achieved using a method adapted from Kumar and Katyal (23). Cells were differentiated with 2% FBS and 20 μM retinoic acid (RA) in DMEM. The differentiation process was conducted over periods of 4, 6, and 8 days, with media refreshment every 2 days. Images were captured using Nikon Eclipse TS100 inverted microscope (magnification 20×). The ImageJ-simple neurite tracer plugin (Image J, version 1.52, NIH) was used to quantify the average neurite length. An average of 10 cells/field were traced and represented in the graph. Briefly, the tracer plugin transformed the images to an 8-bit format for analysis. Neurite tracing was performed manually from the soma junction, following the neurite’s path until reaching the neurite tip. Neurites exceeding twice the soma’s diameter were considered indicative of cell differentiation (23).

### 2.2 Patch-clamp electrophysiology

We also explored the functional properties of the differentiated cells electrophysiologically, by recording membrane potentials and voltage-gated Na^+^ and K^+^ currents. Patch-clamp recordings were performed in whole-cell configuration at room temperature, using an Axopatch 200B amplifier (Axon Instruments, Union City, California) interfaced to a PC with an ITC-18 computer interface (Instrutech Corp, Port Washington, New York). Electrodes were pulled from borosilicate glass capillaries and had resistances of 4–6 MΩ when filled with the internal solution. Seal resistances ranged between 5 and 10 GΩ. The membrane was ruptured by further suction. The extracellular solution contained 125 mM NaCl, 4 mM KCl, 1.2 mM MgSO_4_, 10 mM glucose, and 10 mM HEPES (pH 7.4 with NaOH). The intracellular pipette solutions were prepared following a published protocol by Sahin et al. (24) with certain modifications: 140 mM KCl, 4 mM NaCl, 0.02 mM CaCl_2_, 0.8 mM EGTA, 2 mM MgCl_2_, 4 mM Mg ATP, and 10 mM HEPES (pH 7.4 with KOH) (9). Na^+^ currents were recorded in the presence of CdCl_2_ (0.2 mM), to block Ca^2+^ currents. K^+^ currents were recorded in the presence of both CdCl_2_ (0.2 mM) and TTX (300 nM), to block Ca^2+^ and Na^+^ currents, respectively. Drugs were applied through a gravity-driven perfusion system and a complete exchange of the bath solution occurred in about 2 min. A sampling interval of 10 μs/point and series resistance compensation of 60–70% was applied. Currents were filtered at 5 kHz. Stimulation, acquisition, data analysis, and curve fitting were performed with pCLAMP software (Axon Instruments, Burlin-game, CA) and ORIGIN (Microcal Software, Northampton, MA).

### 2.3 Intermittent theta burst stimulation set-up

**Figure 1:**
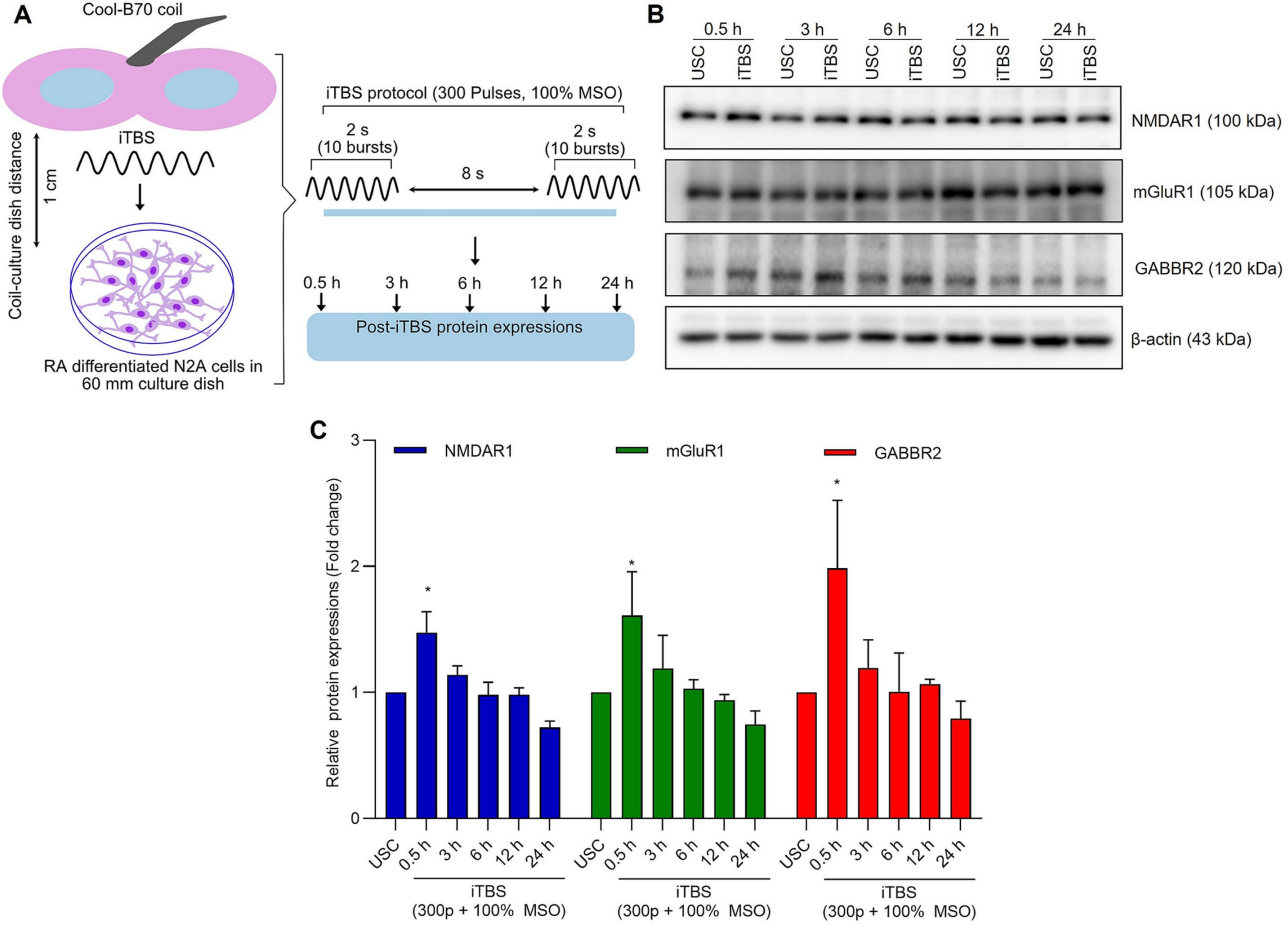
The expression of excitatory and inhibitory receptors subunits over 24 h after a single session of iTBS in RA-differentiated N2A cells. **(A)** Schematic illustration of the experimental protocol. Differentiated N2A cells were stimulated with a single session of iTBS (300 pulses at 100% MSO) using Cool-B70 coil (180 mm × 116 mm/7.1 × 4.6 in), distance between culture dish-coil is 1 cm. **(B)** A representative figure depicts the immunoblot expressions of NMDAR1, mGluR1, and GABBR2 protein levels after 0.5, 3, 6, 12, and 24 h post-iTBS. **(C)** Quantitative data shows the fold change of indicated protein levels relative to β-actin. Band intensity was normalized using β-actin, which also served as the loading control. Quantitative data are shown from three independent experiments (*n* = 3) and are expressed as the mean ± SD [Kruskal–Wallis *H* test, ^*^p < 0.05 vs. unstimulated cells (USC)].

Differentiated N2A cells have previously been utilized to investigate the effects of repetitive magnetic stimulation on the molecular mechanisms behind ischemic/reperfusion injury (25, 26). For stimulation set-up (Figure 1A), we subjected N2A cells to iTBS using a MagPro X100 equipped with a MagOption stimulator. Culture dishes were positioned 1 cm below the center of a Cool-B70 figure-of-8 coil (Magventure, Denmark) to ensure optimal exposure to the magnetic field. The device generates approximately 1.5–2.0 Tesla at the surface of the coil during maximum pulse delivery. The iTBS protocol was modified to deliver 300 pulses in a 2-s train with an 8-s interval between trains. This modification was crucial to maintain the viability of the cells while ensuring effective stimulation. To maintain experimental consistency, all cell groups, including the unstimulated controls, were placed outside the incubator for the same duration. The unstimulated cells were also positioned under the coil for 120 seconds with the stimulator turned on, but without delivering actual stimulation, serving as a baseline comparison for the stimulated groups.

### 2.4 Cytotoxicity assessment

The cytotoxicity of iTBS on differentiated N2A cells was analyzed using WST-8 Cell Proliferation Assay Kit (Biomax Co, Ltd., Seoul, South Korea). N2A cells (1 × 10^4^) were seeded in a 12-well plate and subsequently differentiated using RA for 4 days. The cells were then stimulated with 300 pulses of iTBS at 25, 50, 75 and 100% maximum stimulation output (MSO). At 24 h post-iTBS, cells were incubated with WST-8 reagent for an hour. The difference in absorbance was measured at 450 nm compared to the reagent blank (27).

### 2.5 Immunoblotting

For immunoblotting, we followed established protocols (28). Proteins from all cell groups were extracted using RIPA buffer (Sigma-Aldrich), and their concentrations determined via BCA protein assay (Thermo Fisher). These proteins were then transferred to PVDF membranes. To ensure specificity and sensitivity, we performed titration experiments with primary antibodies targeting key proteins such as Bax, Bcl-2, Caspase-3, NMDAR1, and others, each diluted to optimal concentrations; Bax (Cell Signaling Technology, #4671, 1: 1000), Bcl-2 (Cell Signaling Technology, #9212, 1:1000), Caspase-3 (Cell Signaling Technology, #9211, 1:1000), NMDAR1 (Thermo Fisher, #PA5-85751, 1:1000), mGluR1 (Thermo Fisher, #PA1-46151, 1:1000), GABBR2 (Cell Signaling Technology, #702625, 1:1000), β-tubulin-III (Biolegend, #801201, 1:1000), GAP-43 (Millipore, #AB5220, 1:1000), phosphor-TRKB (Thermo Fisher, #MA5-32207, 1:1000), TRKB (Thermo Fisher, #MA5-14903, 1:1000), synapsin-1 (Thermo Fisher, #51-5200, 1:1000), BDNF (Thermo Fisher, #PA5-95183, 1:1000), MAP-2 (Thermo Fisher, #13-1500, 1:1000), and β-actin (Thermo Fisher, #PA1-183, 1:1000). Post-primary antibody incubation membranes were treated with peroxidase-conjugated secondary antibodies. Detection was achieved using SuperSignal West Pico PLUS Chemiluminescent Substrate (Thermo Fisher). The relative band intensities were quantified using the Image J software (Version 1.52, National Institutes of Health, MD, United States). The ratio of target protein density to the corresponding β-actin signal to normalize the data and account for loading variability. Results were expressed as fold changes in protein density, with the experimental groups normalized to their respective controls at each time point (fold change of 1).

### 2.6 Immunocytochemistry

For Immunocytochemistry, Neuro-2a cells (5 × 10^4^) were cultured in 60 mm Petri dishes on Poly-D-Lysine-coated coverslips and differentiated with retinoic acid (RA). Post-4 days of differentiation, cells were subjected to iTBS (100% MSO, 300 pulses) at various time intervals (0.5, 3, 6, 12, 24 h). Cells were then fixed in 4% paraformaldehyde, blocked with a mixture of normal goat serum, bovine serum albumin, and Triton X-100, and incubated with primary antibodies against NMDAR1 (Thermo Fisher, #PA5-85751, 1:600), β-tubulin-III (Biolegend, #801201, 1:600), GABBR2 (Cell Signaling Technology, #702625, 1:600) and Neurofilament-H (Biolegend, #801701, 1:600) for overnight at 4°C, and then incubated with corresponding Alexa Fluor 488 (Invitrogen, #488-A32731, 1:500) or Alexa Fluor 546 (Invitrogen, # A-11003, 1:500) con-jugated secondary antibodies for 1 hat dark at room temperature. This was followed by incubation with Alexa Fluor conjugated secondary antibodies. After washing, coverslips were mounted using DAPI-containing Vectashield solution (#H-2000, United States) and examined under a Nikon Eclipse Ni fluorescence microscope. Fluorescent intensity analysis was performed using ImageJ, based on a protocol by Jayaraj et al. (29). Three random fields were analyzed for each sample to measure fluorescence intensity, with calculations of total corrected cellular fluorescence (TCCF), and results were presented as corrected total cell fluorescence (CTCF). An observer blinded to treatment conditions conducted all measurements to ensure unbiased results. Fluorescence intensity was normalized to the respective controls using the formula: mean fluorescence intensity (MFI) = MFI of the stimulated population / MFI of the unstimulated population. This calculation was applied for each marker protein at each time point, and the values were expressed as fold changes in intensity relative to the controls, as reported in previous experiments (30).

### 2.7 Statistical analysis

All statistical analyses were performed using GraphPad Prism 8 Software (GraphPad Software Inc., La Jolla, CA, United States). The data are presented as the mean ± standard deviation (SD) of three independent experiments (*n* = 3). For parametric data like [eurite length, action potential (AP)], a one-way ANOVA followed by Dunnett’s tests was performed. For non-parametric data (western blotting and ICC), a Kruskal–Wallis *H* test was performed. Values considered to be statistically significant at ^*^p < 0.05, ^**^p < 0.01, and ^***^p < 0.001; ns represents non-significant.

## 3 Results

### 3.1 A short retinoic acid differentiation protocol yields neuronal-like cells

**Figure 2:**
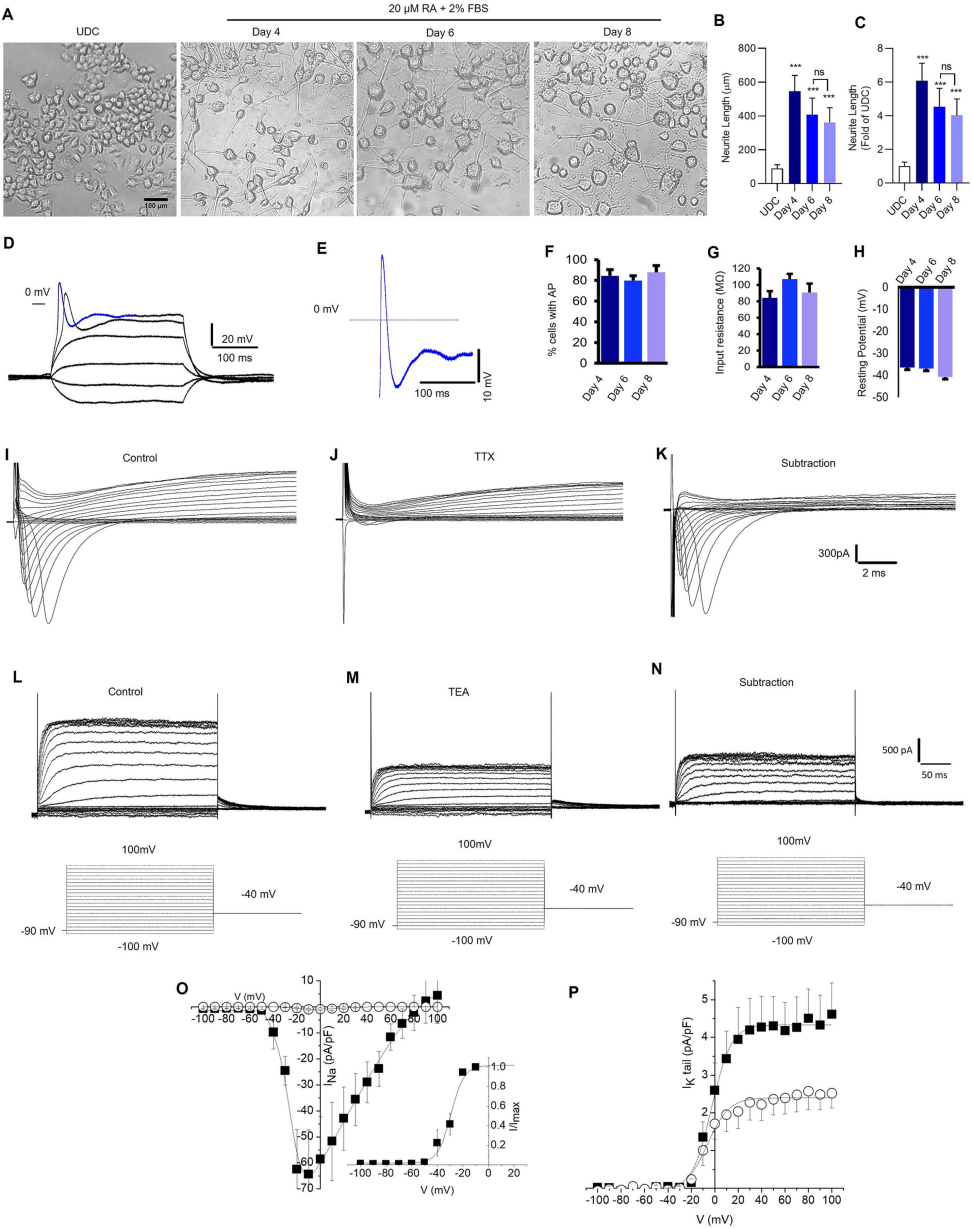
Retinoic acid-differentiated N2A cells exhibit neuronal-like features. **(A)** Representative photomicrograph shows the neurite outgrowth patterns of undifferentiated (UDC) cells [media with 2% FBS and no retinoic acid (RA) (20 μM)] and differentiated N2A cells (media contains 2% FBS and 20 μM RA) for 4, 6, and 8 days (magnification 20×, scale bars: 100 μm). **(B)** A representative bar depicts the average length (μm) of neurite outgrowth in N2A cells after 4, 6, and 8 days of RA treatment. **(C)** The graphical data displays the fold change comparisons of neurite outgrowth in UDC and RA-differentiated N2A cells at 4, 6, and 8 days. The values are expressed as mean ± SD (*n* = 10) and are statistically significant by one-way ANOVA, Dunnett’s test (^***^p = 0.0001 compared with undifferentiated cells (UDC), ns non-significant with day 6 RA-treated N2A cells). **(D,E)** Sample traces showing the recording of action potentials from neuronal-like cells differentiated from N2A cells and carried out in current-clamp mode. **(D)** Membrane potential changes were elicited by applying −0.7 to 2.2 nA, 300 ms pulses (the membrane potential was held at −70 mV). **(E)** Enlargement of the potential resembling an action potential highlighted in **D**, as a blue colored trace (the dashed line indicates 0 mV level). Signals were sampled at 50 kHz and filtered at 5 kHz. **(F)**. The percentage of cells exhibiting action potentials at 4, 6, and 8 days (ns; number of cells = 21, 64, and 20 respectively). **(G)** Input resistance at 4, 6 and 8 days (ns; number of cells = 21, 64, 20 respectively). **(H)** Membrane resting potential at 4, 6 and 8 days (ns, number of cells = 23, 66, 20 respectively). **(I,J)** Representative families of current traces recorded in voltage-clamp mode by applying depolarizing steps from −100 mV to 100 mV (10 mV increment) from a holding potential of −90 mV (the step protocols are reported as insets below current traces), in control conditions **(I)** and after bath application of TTX 300 nM **(J)**. **(K)** Pure Na^+^ current expressed by differentiated N2A cells calculated by subtracting the current recorded in the presence of TTX from control currents. **(L,M)** Representative families of K^+^ current traces recorded in control conditions **(L)** and after bath application of TEA 1 mM **(M)**. **(N)** TEA sensitive K^+^ currents calculated by subtracting the current recorded in the presence of TEA from control currents. **(O)** Current–voltage (IV) relationships of peak Na^+^ current density plotted as a function of step potentials (10 mV increment) and calculated in control conditions (■) and after the superfusion of TTX 300 nM (○). (Inset) Normalized IV data points for the INa activation phase fitted with a Boltzmann relationship. The best fit parameters are control V1/2 = 29.4 mV and *k* = 5.2 (data are mean ± SD, *n* = 10). **(P)** Tail current density recorded at −40 mV and plotted as a function of depolarizing step potentials (10 mV increment) in control conditions (■) and after the superfusion of TEA 1 mM (○). The solid lines represent fits of experimental data points with a Boltzmann relationship. The best fit parameters are control V1/2 = −2.7 mV and *k* = 8.3; TEA 1 mM: V1/2 = −6.0 mV and *k* = 9.1 (data are the mean ± SD, *n* = 6).

As shown in Figures 2A,B, cells treated with RA had significantly longer neurite length compared to undifferentiated N2A cells (*p* < 0.0001). The averaged neurite length after 4-, 6-, and 8-day differentiation protocol was 546.3 ± 93.25 μm, 407.9 ± 97.94 μm, and 361.6 ± 87.56 μm, respectively. The averaged neurite length of undifferentiated N2A cells was 89.9 ± 6.8 μm. The most significant increase in neurite outgrowth was observed following 4-day RA treatment (6.08 ± 1.04-fold, *p* < 0.0001) compared to 6-day (4.54 ± 1.09-fold), 8-day (4.02 ± 0.97-fold) RA treatment (Figure 2C).

Compared to undifferentiated N2A cells, RA differentiated cells had increased expression of neuronal-specific markers, specifically, 1.4-fold change in MAP-2, β-tubulin-III, and GAP-43 (Supplementary Figure S1). To confirm the neuronal properties of the differentiated cells, we recorded action potentials (APs) using patch-clamp technique (Figures 2D,E). The cells were patch-clamped using the whole-cell configuration and APs were elicited in the current clamp mode. The number of cells recorded at 4, 6, and 8 days were 21, 64, and 20, respectively. Approximately 80% of the cells displayed AP after 4, 6, and 8 days of RA treatment (Figure 2F). Both the cell resting membrane potential and input resistance did not change remarkably after RA application for 4–8 days (Figures 2G,H). Depolarizing pulses elicited robust Na^+^ currents, which were completely inhibited by tetrodotoxin (TTX) 300 nM, a specific Na^+^ channel blocker (Figures 2I–K). Furthermore, cells expressed voltage-gated K^+^ channels (Figure 2L), which were partially inhibited by tetraethylammonium (TEA) 1 mM, a K^+^ channel blocker (Figures 2M–P). Overall, the findings suggest that a 4-day RA treatment effectively differentiated N2A cells phenotypically resembling neuronal features.

We evaluated the effect of 300 pulses of iTBS at different stimulation output intensities (25, 50, 75, and 100%) on the viability of these neurons, to exclude the potential cytotoxic effect of iTBS on the differentiated N2A cells. At 24 h post-stimulation, iTBS had no detrimental effect on the viability of N2A cells. We did not find any significant change in the level of expression of pro-apoptotic (Bax, Caspase-3) and anti-apoptotic (Bcl-2) proteins at any MSO conditions, suggesting that 300 pulses of iTBS at maximum intensity (100%) was well tolerated by neurons. Therefore, based on our primary considerations mentioned in the introduction, we concluded that this dose of iTBS is adequate for the remainder of the experiments (Supplementary Figure S2).

### 3.2 iTBS alters the expression of proteins in a time-dependent manner

At 0.5 h post-iTBS, there was a significant increase in the expression of NMDAR1, GABBR2, mGluR1 compared to the unstimulated cells, as confirmed by western blot analysis (*p* < 0.05). The effect on receptor subunits expression was not sustained at later time points (3, 6, 12, 24 h) (Figures 1B,C and Table 1).

**Table 1:** Summary of time-dependent protein expressions induced by iTBS in RA-differentiated N2A cells using western blotting and immunocytochemistry.

Proteins	iTBS (300 pulses at 100% MSO)				
	*H*				
	0.5	3	6	12	24
Western blotting					
NMDAR1	↑^*^	±^ns^	↓^ns^	±^ns^	↓^ns^
GABBR2	↑^*^	↑^ns^	↓^ns^	↑^ns^	↓^ns^
mGluR1	↑^*^	↑^ns^	↓^ns^	±^ns^	↓^ns^
Phospho-TRKB/TRKB	↑^*^	↓^ns^	↓^ns^	↑^ns^	±^ns^
GAP-43	↑^*^	↑^ns^	±^ns^	±^ns^	↓^ns^
Synapsin-1	↑^*^	±^ns^	±^ns^	↑^ns^	↓^ns^
BDNF	↑^*^	↑^ns^	↓^ns^	↑^ns^	↓^ns^
β-tubulin III	↑^*^	↑^ns^	↓^ns^	↑^ns^	↓^ns^
β-actin	±^ns^	±^ns^	±^ns^	±^ns^	±^ns^
Immunocytochemistry					
NMDAR1	↓^ns^	↑^ns^	↑^ns^	↓^ns^	↑^ns^
GABBR2	↓^ns^	↑^ns^	↓^ns^	↓^ns^	↓^ns^
β-tubulin III	↓^ns^	↑^ns^	↑^ns^	↓^ns^	↑^ns^
Neurofilament-H	↓^ns^	↑^ns^	↓^ns^	↓^ns^	↓^ns^

Values are the representatives of three independent experiments and are statistically significant by Kruskal–Wallis *H* test. Comparisons: ^*^p < 0.05, and ns-non-significant with respective unstimulated control at each time point. H, hour; ↑, upregulation; ↓, downregulation; ±, no difference; iTBS, intermittent theta burst stimulation; NMDAR1, N-methyl-D-aspartate receptor; GABBR2, γ-aminobutyric acid type B receptor subunit 2; mGluR1, metabotropic glutamate receptor 1; TRKB, tropomyosin receptor kinase B; GAP-43, growth-associated protein-43; BDNF, brain-derived neurotrophic factor.

**Figure 3:**
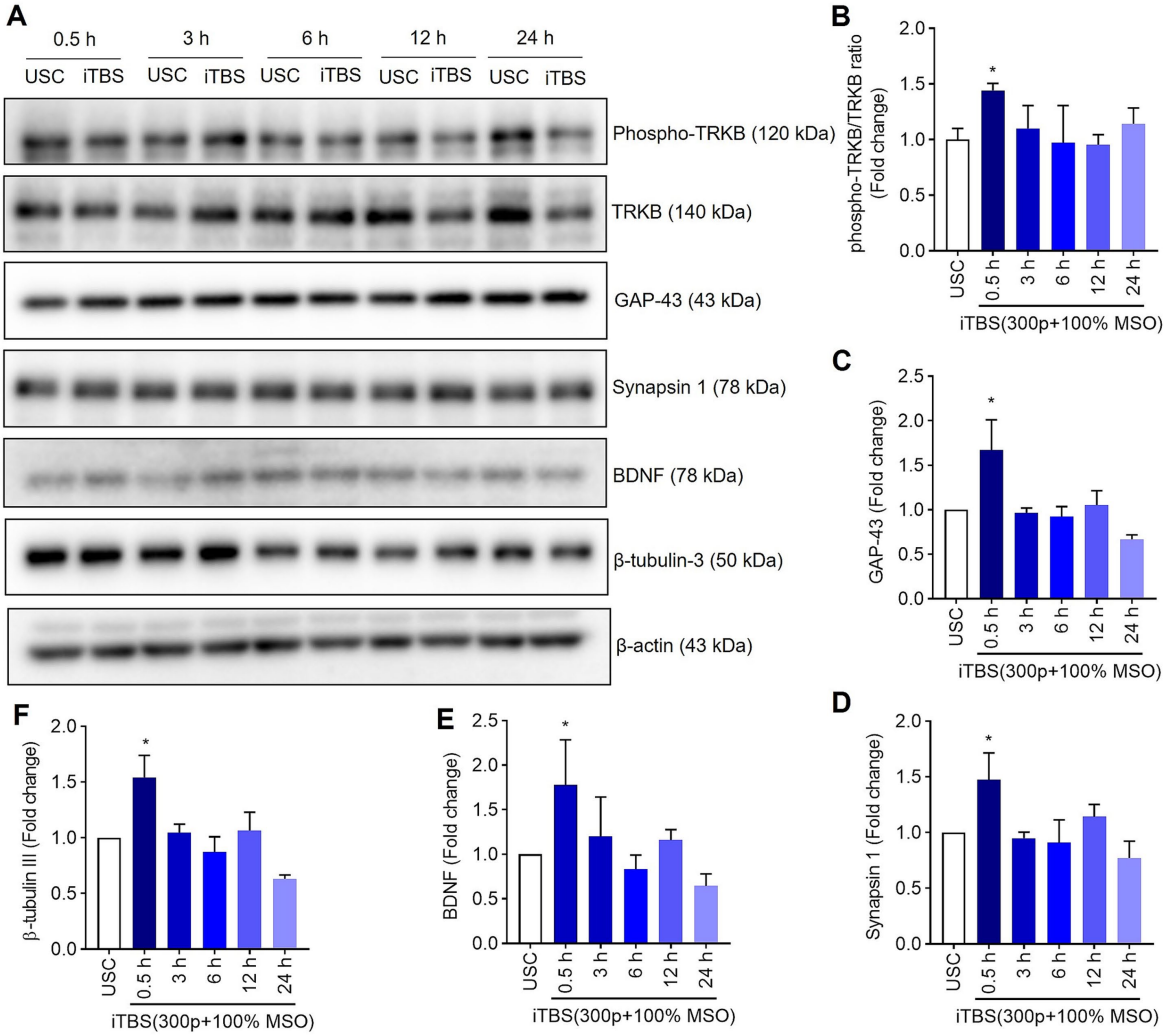
The expression of neural associated proteins over 24 h after a single session of iTBS in RA-differentiated N2A cells. **(A)** A representative immunoblot depicts the protein levels of phospho-TRKB/TRKB, GAP-43, synapsin-1, BDNF, and β-tubulin III protein levels after 0.5, 3, 6, 12, and 24 h post iTBS. **(B–F)** Quantitative data shows the fold change of indicated protein levels relative to β-actin. Band intensity was normalized using β-actin, which also served as the loading control. Quantitative data are shown from three independent experiments (*n* = 3) and are expressed as the mean ± SD [Kruskal–Wallis *H* test, ^*^p < 0.05 vs. unstimulated cells (USC)].

**Figure 4:**
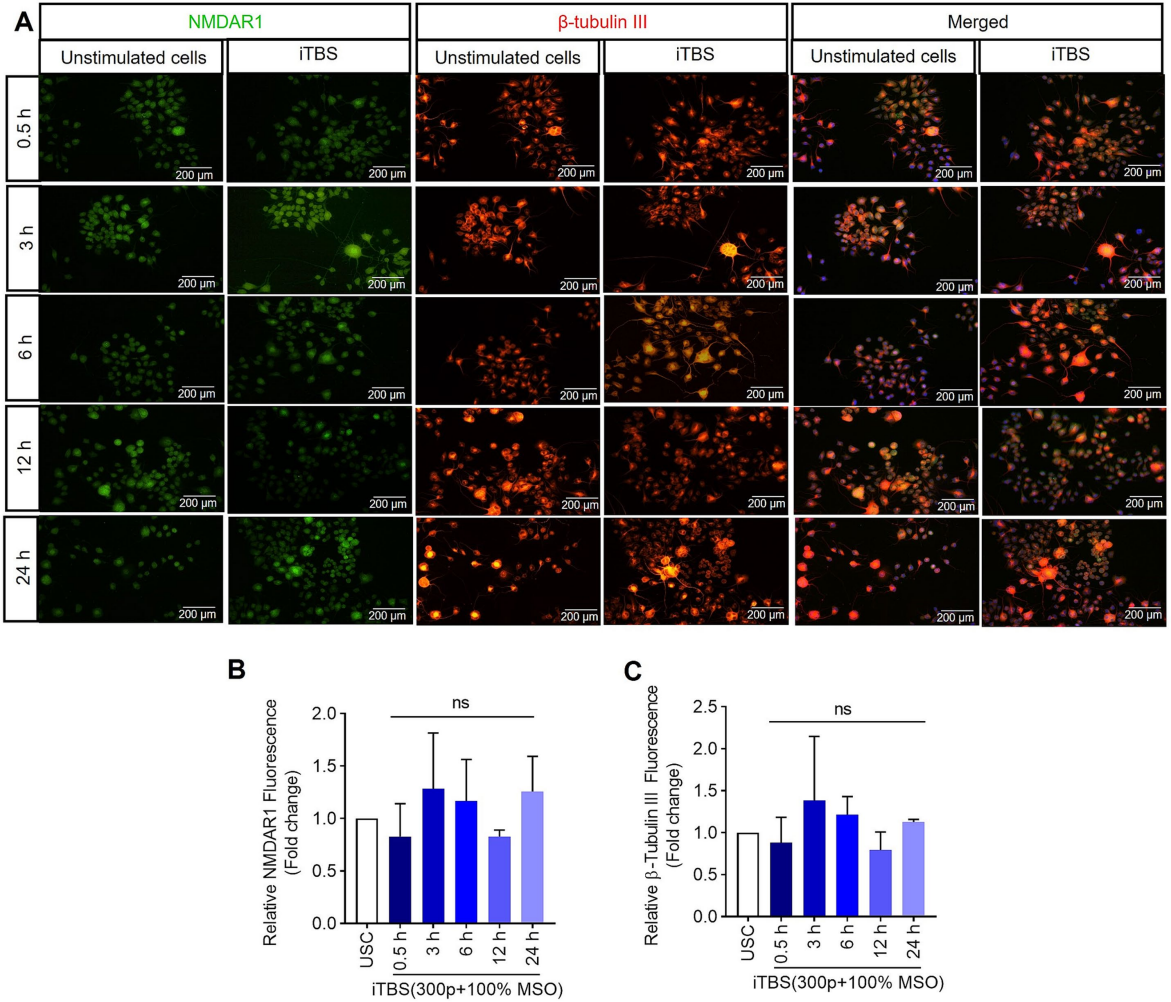
The expression of NMDAR1 and β-tubulin III over 24 h after a single session of iTBS in RA-differentiated N2A cells. **(A)** A representative figure shows the immunofluorescence staining for NMDAR1 (green, Alexa Fluor 488) and β-tubulin III (orange, Alexa Fluor 546) after 0.5, 3, 6, 12, and 24 h post-iTBS in RA-differentiated N2A cells (magnification 10×, scale bars: 200 μm). **(B,C)** Quantification of mean fluorescence intensity (MFI), presented as fold change are shown from three independent experiments [mean ± SD, *n* = 3, Kruskal–Wallis *H* test, ns-not significant with unstimulated cells (USC)].

**Figure 5:**
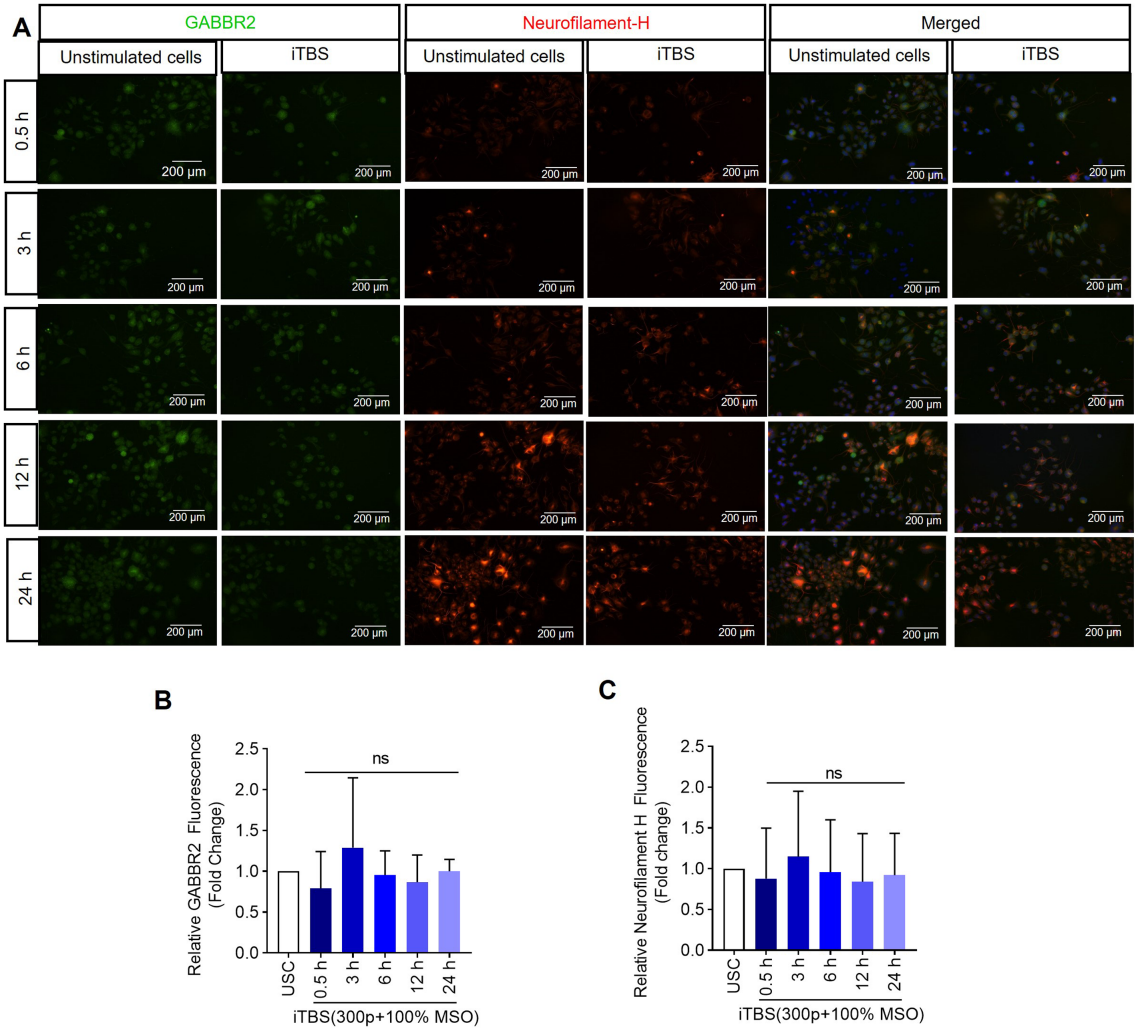
The expression of GABBR2 and Neurofilament-H over 24 h following a single dose of iTBS. **(A)** A representative image depicts the immunofluorescence staining for GABBR2 (green, Alexa Fluor 488) and Neurofilament-H (orange, Alexa Fluor 546) after 0.5, 3, 6, 12, and 24 h post-iTBS in RA-differentiated N2A cells (magnification 10×, scale bars: 200 μm). **(B,C)** Quantification of mean fluorescence intensity (MFI), presented as fold change are shown from three independent experiments (mean ± SD, *n* = 3). Statistical significances were calculated and compared with unstimulated cells (USC) of each respective time points by Kruskal–Wallis *H* test (ns, not significant compared with USC).

Similarly, at 0.5 h post-iTBS, there was a significant increase in the expression of phospho-TRKB/total-TRKB, GAP-43, synapsin-1, BDNF and β-tubulin III compared to the unstimulated cells (*p* < 0.05). However, the effect on neurotrophic factors, and cytoskeletal proteins was not sustained at later time points (Figure 3 and Table 1). When analyzed using immunocytochemistry (ICC), the expression of NMDAR1 and β-tubulin III (Figures 4A–C and Table 1) and the expression GABBR2 and Neurofilament-H (Figures 5A–C and Table 1) initially decreased at 0.5 h and then increased at 3 h. However, these changes were not statistically significant compared to unstimulated cells. The effect was variable and not significant at later timepoints.

## 4 Discussion

Given the promising clinical applications of iTBS, understanding its cellular and molecular mechanisms is essential for designing effective treatment protocols (31, 32). In this study, we investigated the changes in expression of selected neurotransmitter receptor subunits, neurotrophic factors, and cytoskeletal proteins induced by iTBS in differentiated N2A cells. Our results show that iTBS elicits rapid alterations in the expression of all selected proteins, occurring within 0.5 h post stimulation. These acute changes are unlikely due to methodological error, as the expression levels was normalized to the housekeeping protein (β-actin). Rather, the immediate response suggests the potential of iTBS to rapidly influence gene expression, similar to the reported effects of non-patterned repeated rTMS, which have been linked to mechanisms like histone acetylation modulation (33). Additionally, such rapid gene expression changes are consistent with studies showing that rTMS can affect cortical gene expression in models of cerebral ischemia-reperfusion injury and contribute to recovery processes (34, 35). Another potential mechanism may involve stimulation-induced redistribution of receptors within the cell, leading to a higher receptor concentration at the synaptic level. This could create a false impression of increased expression when analyzing lysates from whole cells via western blot, as done in this study. Lastly, we cannot exclude the possibility that the observed increase reflects new protein synthesis due to activity-related changes in gene expression. This is supported by studies using electrical stimulation of neurons, which have shown an average transcriptional delay of 10–20 min before mRNA production, with proteins being expressed and detected within 20–30 min of stimulation (13). Whether similar mechanisms are triggered following a single train of iTBS at a lower intensity (total dose) is an important question requiring future investigation.

The results also show a pronounced upregulation of key proteins such as phospho-TrkB/TrkB, GAP-43, BDNF, and synapsin-1 within the 0.5 h post-iTBS, mirroring the early expression patterns observed in primary neurons. This significant elevation, particularly of phospho-TrkB/TrkB and BDNF at 0.5 h supports the pivotal role of the BDNF-TrkB signaling pathway in mediating synaptic plasticity and promoting neurite outgrowth. This activation aligns with previous findings, emphasizing the pathway’s involvement in the consolidation phase of synaptic plasticity, which occurs within minutes to hours after stimulation (17, 36). The concurrent increase in GAP-43 and synapsin-1 levels further suggests an iTBS-driven modulation of synaptic vesicle dynamics and neurite extension. The subsequent normalization of these protein levels may reflect inherent compensatory responses aimed at preserving synaptic equilibrium or it may indicate the temporary nature of their contributions to iTBS-induced plasticity following a single application. We cannot rule out the possibility that prestored soluble factors released by neurons into the media following iTBS may contribute to the transient increase in protein expression observed at the 0.5 h time point. Future studies will aim to sample the media at multiple timepoints to identify these factors.

To further investigate the effects of iTBS on synaptic plasticity markers, we expanded our study to include cytoskeletal proteins, observing a notable pattern in synapsin-1 and β-tubulin III expression. Synapsin-1, crucial for neurotransmitter release and synaptic vesicle trafficking, showed an increase at 0.5 h post-iTBS, paralleling the enhanced expression of neurotransmitter receptors. This synchrony underscores the coordinated modulation of synaptic function and structural dynamics shortly after iTBS. In contrast, while we detected an upsurge in β-tubulin III at 0.5 h, suggesting microtubule involvement in early response to iTBS, Thomson et al. (14) did not observe significant changes in β-tubulin III at later time points in a similar context. This discrepancy may reflect differences in temporal expression patterns and experimental conditions between studies.

The absence of significant changes in Neurofilament-H suggests either a nuanced effect of iTBS on neuronal cytoskeletal stability and axonal integrity at the applied dose or the need for longer assessments to discern iTBS’s influence on cytoskeletal components. These findings, coupled with methodological differences between gene expression and protein analysis techniques, highlight the complexity of interpreting iTBS-induced molecular dynamics. Future comparative studies across multiple time points and stimulation protocols are crucial to gain better understanding of iTBS’s effects on the neuronal cytoskeleton and broader molecular mechanisms underlying brain stimulation protocols.

The configuration of iTBS protocols significantly influences their neuromodulatory effects. Clinical studies have documented the critical role of the interval between iTBS sessions in determining the overall outcome of neuromodulation (37). For example, Yu et al. (38) found that a single iTBS train delivered at 70% active motor threshold (AMT) with 1800 pulses inhibited motor evoked potentials (MEP), whereas distributing iTBS into multiple sessions enhanced MEP amplitude, indicating a sustained effect. Similarly, Tse et al. (39) found that the interval between iTBS trains could modulate MEP outcomes, with shorter intervals leading to a reduction (LTD-like effect) and longer intervals facilitating MEP (LTP-like effect). Wang et al. (15) further demonstrated the dose- and time-dependent effects of iTBS in a parkinsonian rat model, compairing single and multiple train protocols. Our findings suggest that even a single train of iTBS comprising only 300 pulses can elicit effects comparable to those observed in longer protocols with varied intervals, aligning with observations by Ljubisavljevic et al. (40) on AMPA RNA levels and by Lee et al. (36) on changes in excitatory and inhibitory synaptic functions following chronic iTBS treatment. These results emphasize the importance of understanding how protocol design, dose, and timing influence the response to iTBS, and highlight the need for further research to optimize these parameters for therapeutic applications.

In our study, differentiated N2A cells not only exhibited structural characteristics akin to neurons but also demonstrated electrophysiological properties indicative of neuronal-like functionality. This assertion is supported by their ability to exhibit action potentials comparable to neurons, alongside the presence of TTX-sensitive voltage-gated sodium currents and TEA-sensitive voltage-gated delayed-rectifier potassium currents, underscoring the robust neuron-like properties of the recorded cells. While N2A cells serve as a validated model for neuronal behavior, it is important to acknowledge that they may not fully encompass the complexity of neurons within brain networks. Nevertheless, this model remains instrumental in identifying dynamic molecular targets, offering valuable insights for future mechanistic studies in both normal and disease contexts. Notably, the use of specific neuronal markers such as MAP-2, β-tubulin III, and GAP-43 further validated the effectiveness of RA-induced differentiation. Our findings align with established research, reinforcing the utility of N2A cells as a reliable model for examining neuronal properties and behaviors.

## 5 Conclusion

Our findings emphasize the importance of exploring the temporal dynamics underlying iTBS-induced neuroplasticity and inter-individual response variability. Such investigations are crucial for optimizing accelerated TMS protocols, particularly in the treatment of psychiatric conditions like depression, where precise modulation of session intervals and dose accumulation could significantly affect therapeutic outcomes. Developing a robust experimental framework for understanding stimulation timing will allow us to identify specific molecular targets and tailor TMS strategies more effectively, paving the way for more personalized and efficacious interventions.

## Data Availability

The raw data supporting the conclusions of this article will be made available by the authors, without undue reservation.

## Supplementary material

SUPPLEMENTARY FIGURE S1 RA-treated N2A cells showed enhanced expression of neuronal-specific markers. **(A)** A representative immunoblots of MAP-2, β-tubulin III, and GAP-43 expressions in undifferentiated cells (UDC, media with 2% FBS) and differentiated N2A cells [media contains 2% FBS and 20 μM retinoic acid (RA)] for 4, 6, and 8 days. **(B)** Quantitative data shows the fold change of indicated protein levels relative to β-actin. Band intensity was normalized using β-actin, which also served as the loading control. Quantitative data are shown from three independent experiments (*n* = 3) and are expressed as the mean ± SD (Kruskal–Wallis *H* test, ^*^*p* < 0.05 vs. UDC).

SUPPLEMENTARY FIGURE S2 **(A)** Experimental design for iTBS stimulation. N2A cells were differentiated with retinoic acid (RA, 20 μM) for 4 days, followed by iTBS stimulation using C-B70 coil (300 pulses at different stimulator output (SO) intensities: 25%, 50%, 75%, and 100%) for 24 h. **(B)** SO-dependent cytotoxic effects of iTBS on N2A cells at 24 h after stimulation (mean ± SD, *n* = 3), (Kruskal–Wallis *H* test, ns, not significant vs. control cells). **(C)** Immunoblots for apoptosis signaling proteins (Caspase-3, Bax, Bcl-2) on culture of RA differentiated N2A cells at 24 h post-iTBS stimulation. β-actin was used as an internal control. **(D)** Bar graph shows the densitometry analysis of respective proteins, quantified by ImageJ. The values are expressed as the mean ± SD of three independent experiments (Kruskal–Wallis *H* test, ns, not significant vs. control cells).

## References

[1] Kumar S Ferraro M Nguyen L Cao N Ung N Jose JS. TMS assessment of corticospinal tract integrity after stroke: broadening the concept to inform neurorehabilitation prescription. Front Hum Neurosci. (2024) 18:1408818. doi: 10.3389/fnhum.2024.1408818, PMID: 39290568 PMC11405325

[2] Sondergaard RE Martino D Kiss ZHT Condliffe EG. TMS motor mapping methodology and reliability: a structured review. Front Neurosci. (2021) 15:709368. doi: 10.3389/fnins.2021.709368, PMID: 34489629 PMC8417420

[3] Liao WY Opie GM Ziemann U Semmler JG. The effects of intermittent theta burst stimulation over dorsal premotor cortex on primary motor cortex plasticity in young and older adults. Eur J Neurosci. (2024) 60:4019–33. doi: 10.1111/ejn.16395, PMID: 38757748

[4] Maiella M Casula EP Borghi I Assogna M D’Acunto A Pezzopane V. Simultaneous transcranial electrical and magnetic stimulation boost gamma oscillations in the dorsolateral prefrontal cortex. Sci Rep. (2022) 12:19391. doi: 10.1038/s41598-022-23040-z, PMID: 36371451 PMC9653481

[5] Huang YZ Edwards MJ Rounis E Bhatia KP Rothwell JC. Theta burst stimulation of the human motor cortex. Neuron. (2005) 45:201–6. doi: 10.1016/j.neuron.2004.12.033 15664172

[6] Gamboa OL Antal A Moliadze V Paulus W. Simply longer is not better: reversal of theta burst after-effect with prolonged stimulation. Exp Brain Res. (2010) 204:181–7. doi: 10.1007/s00221-010-2293-4, PMID: 20567808 PMC2892066

[7] Amer A Martin JH. Repeated motor cortex theta-burst stimulation produces persistent strengthening of corticospinal motor output and durable spinal cord structural changes in the rat. Brain Stimul. (2022) 15:1013–22. doi: 10.1016/j.brs.2022.07.005, PMID: 35850438 PMC10164459

[8] Bian L Zhang L Huang G Song D Zheng K Xu X. Effects of priming intermittent theta burst stimulation with high-definition tDCS on upper limb function in hemiparetic patients with stroke: a randomized controlled study. Neurorehabil Neural Repair. (2024) 38:268–78. doi: 10.1177/15459683241233259, PMID: 38357884

[9] Lee KZ Vinit S. Modulatory effect of trans-spinal magnetic intermittent theta burst stimulation on diaphragmatic activity following cervical spinal cord contusion in the rat. Spine J. (2024) 24:352–72. doi: 10.1016/j.spinee.2023.09.013 37774983

[10] Torres IJ Ge R McGirr A Vila-Rodriguez F Ahn S Basivireddy J. Effects of intermittent theta-burst transcranial magnetic stimulation on cognition and hippocampal volumes in bipolar depression. Dialogues Clin Neurosci. (2023) 25:24–32. doi: 10.1080/19585969.2023.2186189, PMID: 36924413 PMC10026761

[11] Zheng B Chen J Cao M Zhang Y Chen S Yu H. The effect of intermittent theta burst stimulation for cognitive dysfunction: a meta-analysis. Brain Inj. (2024) 38:675–86. doi: 10.1080/02699052.2024.2344087, PMID: 38651344

[12] Lin H Liang J Wang Q Shao Y Song P Li S. Effects of accelerated intermittent theta-burst stimulation in modulating brain of Alzheimer’s disease. Cereb Cortex. (2024) 34:bhae106. doi: 10.1093/cercor/bhae106, PMID: 38517175

[13] Lee PR Fields RD. Activity-dependent gene expression in neurons. Neuroscientist. (2021) 27:355–66. doi: 10.1177/1073858420943515, PMID: 32727285 PMC8246373

[14] Thomson AC Kenis G Tielens S de Graaf TA Schuhmann T Rutten BPF. Transcranial magnetic stimulation-induced plasticity mechanisms: TMS-related gene expression and morphology changes in a human neuron-like cell model. Front Mol Neurosci. (2020) 13:528396. doi: 10.3389/fnmol.2020.528396, PMID: 33192288 PMC7604533

[15] Wang Y Liu J Hui Y Wu Z Wang L Wu X. Dose and time-dependence of acute intermittent theta-burst stimulation on hippocampus-dependent memory in parkinsonian rats. Front Neurosci. (2023) 17:1124819. doi: 10.3389/fnins.2023.1124819, PMID: 36866328 PMC9972116

[16] Zhu L Wang Y Lin X Zhao X Fu ZJ. Effects of ozone on Hippocampus BDNF and Fos expressions in rats with chronic compression of dorsal root ganglia. Biomed Res Int. (2021) 2021:5572915. doi: 10.1155/2021/5572915, PMID: 34869766 PMC8642004

[17] Huang YZ Lu MK Antal A Classen J Nitsche M Ziemann U. Plasticity induced by non-invasive transcranial brain stimulation: a position paper. Clin Neurophysiol. (2017) 128:2318–29. doi: 10.1016/j.clinph.2017.09.007, PMID: 29040922

[18] Liu JL Wang S Chen ZH Wu RJ Yu HY Yang SB. The therapeutic mechanism of transcranial iTBS on nerve regeneration and functional recovery in rats with complete spinal cord transection. Front Immunol. (2023) 14:1153516. doi: 10.3389/fimmu.2023.1153516, PMID: 37388732 PMC10306419

[19] Zeljkovic Jovanovic M Stanojevic J Stevanovic I Stekic A Bolland SJ Jasnic N. Intermittent theta burst stimulation improves motor and behavioral dysfunction through modulation of NMDA receptor subunit composition in experimental model of Parkinson’s disease. Cells. (2023) 12:1525. doi: 10.3390/cells12111525, PMID: 37296646 PMC10252812

[20] Nettekoven C Volz LJ Kutscha M Pool EM Rehme AK Eickhoff SB. Dose-dependent effects of Theta burst rTMS on cortical excitability and resting-state connectivity of the human motor system. J Neurosci. (2014) 34:6849–59. doi: 10.1523/JNEUROSCI.4993-13.2014, PMID: 24828639 PMC4019799

[21] Gao B Wang Y Zhang D Wang Z Wang Z. Intermittent theta-burst stimulation with physical exercise improves poststroke motor function: a systemic review and meta-analysis. Front Neurol. (2022) 13:964627. doi: 10.3389/fneur.2022.964627, PMID: 36110393 PMC9468864

[22] Kirkovski M Donaldson PH Do M Speranza BE Albein-Urios N Oberman LM. A systematic review of the neurobiological effects of theta-burst stimulation (TBS) as measured using functional magnetic resonance imaging (fMRI). Brain Struct Funct. (2023) 228:717–49. doi: 10.1007/s00429-023-02634-x, PMID: 37072625 PMC10113132

[23] Kumar M Katyal A. Data on retinoic acid and reduced serum concentration induced differentiation of Neuro-2a neuroblastoma cells. Data Brief. (2018) 21:2435–40. doi: 10.1016/j.dib.2018.11.097, PMID: 30547071 PMC6282191

[24] Sahin M Oncu G Yilmaz MA Ozkan D Saybasili H. Transformation of Sh-Sy5y cell line into neuron-like cells: investigation of electrophysiological and biomechanical changes. Neurosci Lett. (2021) 745:135628. doi: 10.1016/j.neulet.2021.135628 33440235

[25] Baek A Kim JH Pyo S Jung JH Park EJ Kim SH. The differential effects of repetitive magnetic stimulation in an *in vitro* neuronal model of ischemia/reperfusion injury. Front Neurol. (2018) 9:50. doi: 10.3389/fneur.2018.00050, PMID: 29487560 PMC5816832

[26] Baek A Park EJ Kim SY Nam BG Kim JH Jun SW. High-frequency repetitive magnetic stimulation enhances the expression of brain-derived neurotrophic factor through activation of Ca^2+^-calmodulin-dependent protein kinase II-camp-response element-binding protein pathway. Front Neurol. (2018) 9:285. doi: 10.3389/fneur.2018.00285, PMID: 29867712 PMC5949612

[27] Krishnan M Choi J Jang A Kim YA. Novel peptide antibiotic, Pro10-1D, designed from insect defensin shows antibacterial and anti-inflammatory activities in sepsis models. Int J Mol Sci. (2020) 21:6216. doi: 10.3390/ijms21176216, PMID: 32867384 PMC7504360

[28] Dittadi R Catozzi L Gion M Brazzale A Capitanio G Gelli MC. Comparison between western blotting, immunohistochemical and ELISA assay for p185neu quantitation in breast cancer specimens. Anticancer Res. (1993) 13:1821–4. PMID: 7903522

[29] Jayaraj RL Beiram R Azimullah S Nagoor Meeran MF Ojha S Adem A. Noscapine prevents rotenone-induced neurotoxicity: involvement of oxidative stress, neuroinflammation and autophagy pathways. Molecules. (2021) 26:4627. doi: 10.3390/molecules26154627, PMID: 34361780 PMC8348109

[30] Thankam FG Wilson VED Radwan MM Siddique A Agrawal DK. Involvement of ischemia-driven 5-lipoxygenase-resolvin-E1-chemokine like receptor-1 axis in the resolution of post-coronary artery bypass graft inflammation in coronary arteries. Mol Biol Rep. (2022) 49:3123–34. doi: 10.1007/s11033-022-07143-4, PMID: 35061143

[31] Lopez-Alonso V Cheeran B Rio-Rodriguez D Fernandez-Del-Olmo M. Inter-individual variability in response to non-invasive brain stimulation paradigms. Brain Stimul. (2014) 7:372–80. doi: 10.1016/j.brs.2014.02.004, PMID: 24630849

[32] Schilberg L Schuhmann T Sack AT. Interindividual variability and intraindividual reliability of intermittent theta burst stimulation-induced neuroplasticity mechanisms in the healthy brain. J Cogn Neurosci. (2017) 29:1022–32. doi: 10.1162/jocn_a_01100, PMID: 28129054

[33] Etievant A Manta S Latapy C Magno LA Fecteau S Beaulieu JM. Repetitive transcranial magnetic stimulation induces long-lasting changes in protein expression and histone acetylation. Sci Rep. (2015) 5:16873. doi: 10.1038/srep16873, PMID: 26585834 PMC4653621

[34] Feng HL Yan L Cui LY. Effects of repetitive transcranial magnetic stimulation on adenosine triphosphate content and microtubule associated protein-2 expression after cerebral ischemia-reperfusion injury in rat brain. Chin Med J. (2008) 121:1307–12. doi: 10.1097/00029330-200807020-00012 18713553

[35] Boonzaier J van Tilborg GAF Neggers SFW Dijkhuizen RM. Noninvasive brain stimulation to enhance functional recovery after stroke: studies in animal models. Neurorehabil Neural Repair. (2018) 32:927–40. doi: 10.1177/1545968318804425, PMID: 30352528 PMC6238175

[36] Lee CW Chu MC Wu HF Chung YJ Hsieh TH Chang CY. Different synaptic mechanisms of intermittent and continuous theta-burst stimulations in a severe foot-shock induced and treatment-resistant depression in a rat model. Exp Neurol. (2023) 362:114338. doi: 10.1016/j.expneurol.2023.114338, PMID: 36717014

[37] Muller-Dahlhaus F Ziemann U. Metaplasticity in human cortex. Neuroscientist. (2015) 21:185–202. doi: 10.1177/1073858414526645 24620008

[38] Yu F Tang X Hu R Liang S Wang W Tian S. The after-effect of accelerated intermittent theta burst stimulation at different session intervals. Front Neurosci. (2020) 14:576. doi: 10.3389/fnins.2020.00576 32670006 PMC7330092

[39] Tse NY Goldsworthy MR Ridding MC Coxon JP Fitzgerald PB Fornito A. The effect of stimulation interval on plasticity following repeated blocks of intermittent theta burst stimulation. Sci Rep. (2018) 8:8526. doi: 10.1038/s41598-018-26791-w, PMID: 29867191 PMC5986739

[40] Ljubisavljevic MR Javid A Oommen J Parekh K Nagelkerke N Shehab S. The effects of different repetitive transcranial magnetic stimulation (rTMS) protocols on cortical gene expression in a rat model of cerebral ischemic-reperfusion injury. PLoS One. (2015) 10:e0139892. doi: 10.1371/journal.pone.0139892, PMID: 26431529 PMC4592250

