# Supplementary figures and images for: Time dependent changes in protein expression induced by intermittent theta burst stimulation in a cell line
Source: Front Neurol. 2024 Oct 28;15:1396776. doi: 10.3389/fneur.2024.1396776 (PMC11551774; doi:10.3389/fneur.2024.1396776)

SUPPLEMENTARY FIGURE S1

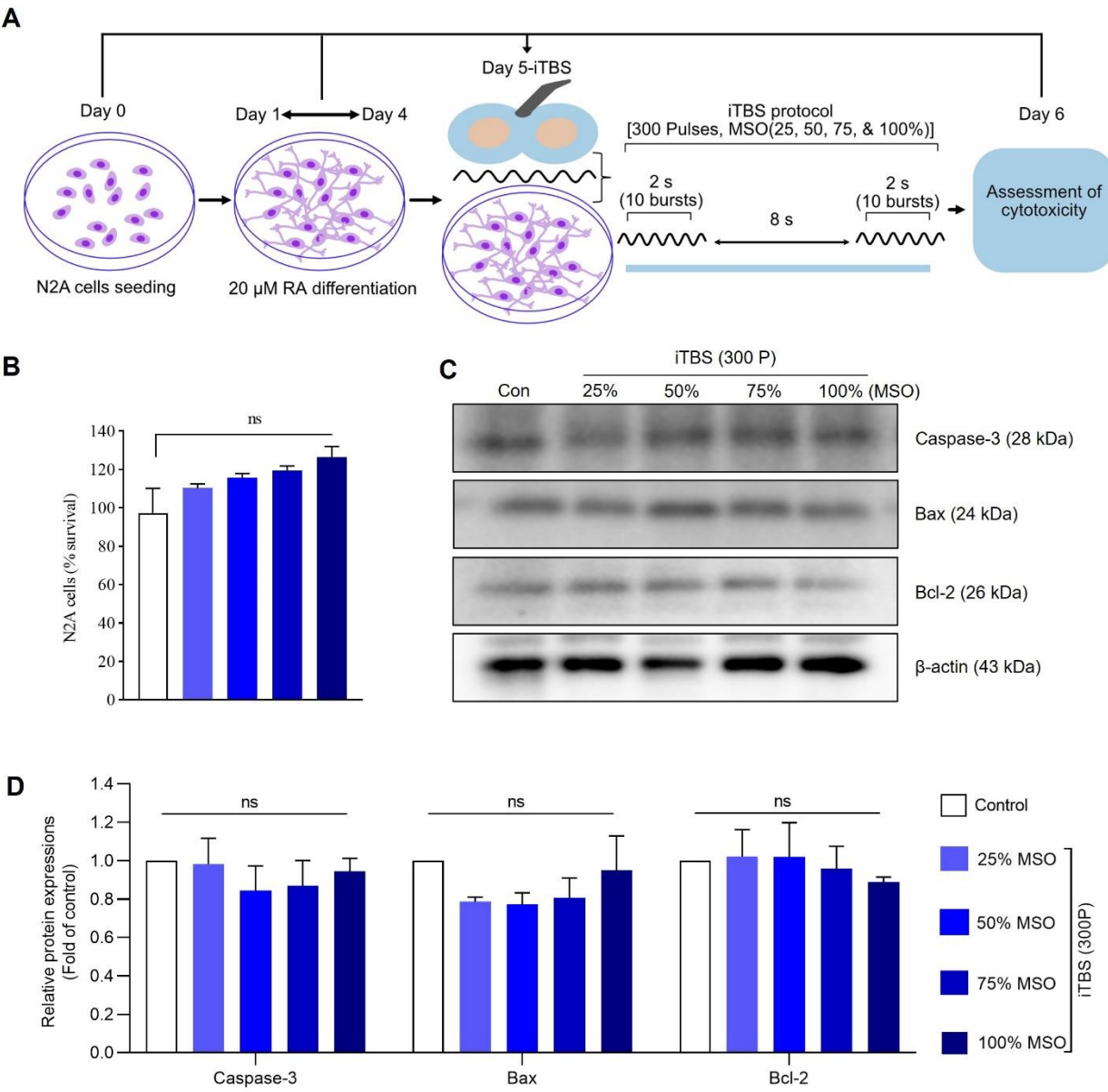

Supplement: SUPPLEMENTARY FIGURE S1 — RA-treated N2A cells showed enhanced expression of neuronal-specific markers. (A) A representative immunoblots of MAP-2, β-tubulin III, and GAP-43 expressions in undifferentiated cells (UDC, media with 2% FBS) and differentiated N2A cells [media contains 2% FBS and 20 μM retinoic acid (RA)] for 4, 6, and 8 days. (B) Quantitative data shows the fold change of indicated protein levels relative to β-actin. Band intensity was normalized using β-actin, which also served as the loading control. Quantitative data are shown from three independent experiments (n = 3) and are expressed as the mean ± SD (Kruskal–Wallis H test, *p < 0.05 vs. UDC). [file Data_Sheet_2.pdf]

SUPPLEMENTARY FIGURE S2

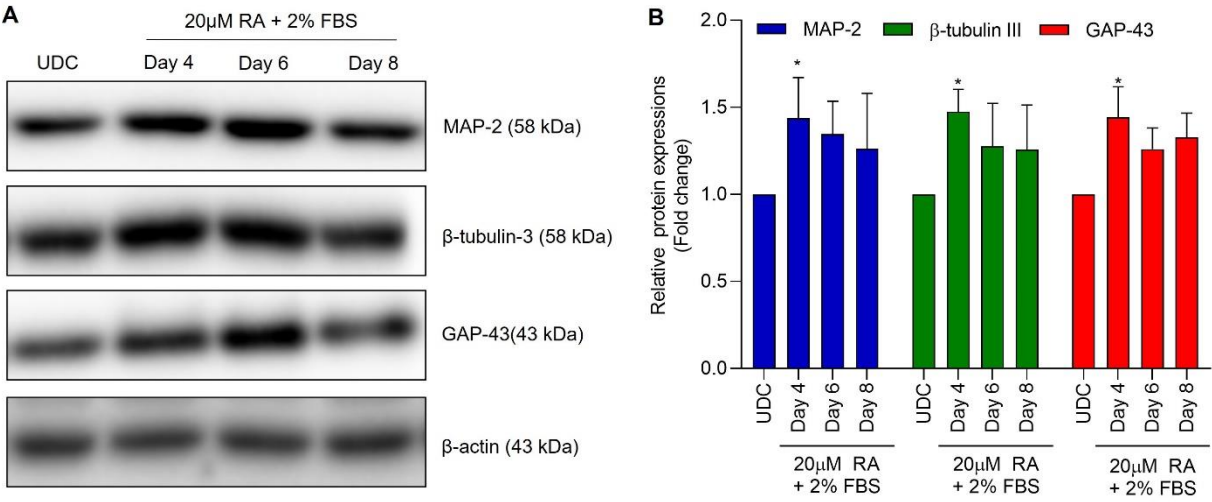

Supplement: SUPPLEMENTARY FIGURE S2 — (A) Experimental design for iTBS stimulation. N2A cells were differentiated with retinoic acid (RA, 20 μM) for 4 days, followed by iTBS stimulation using C-B70 coil (300 pulses at different stimulator output (SO) intensities: 25%, 50%, 75%, and 100%) for 24 h. (B) SO-dependent cytotoxic effects of iTBS on N2A cells at 24 h after stimulation (mean ± SD, n = 3), (Kruskal–Wallis H test, ns, not significant vs. control cells). (C) Immunoblots for apoptosis signaling proteins (Caspase-3, Bax, Bcl-2) on culture of RA differentiated N2A cells at 24 h post-iTBS stimulation. β-actin was used as an internal control. (D) Bar graph shows the densitometry analysis of respective proteins, quantified by ImageJ. The values are expressed as the mean ± SD of three independent experiments (Kruskal–Wallis H test, ns, not significant vs. control cells). [file Data_Sheet_3.pdf]
